# No detection of SARS-CoV-2 in animals exposed to infected keepers: results of a COVID-19 surveillance program

**DOI:** 10.2144/fsoa-2021-0038

**Published:** 2021-04-20

**Authors:** Pellegrino Cerino, Carlo Buonerba, Gianfranco Brambilla, Luigi Atripaldi, Maria Tafuro, Denise Di Concilio, Lucia Vassallo, Gabriella Lo Conte, Maria Concetta Cuomo, Ivana Maiello, Jacopo D'Auria, Davide Cardinale, Maurizio Viscardi, Giuseppe Rofrano, Alfonso Gallo, Pasquale Brusco, Antonio Pizzolante, Vittorio Cicalese, Pio Galdi, Lydia Galdi, Sabato De Vita, Palmiero Volzone, Gabriele Di Vuolo, Annachiara Coppola, Biancamaria Pierri, Giovanna Fusco

**Affiliations:** ^1^Centro di Referenza Nazionale per l'Analisi e Studio di Correlazione tra Ambiente, Animale e Uomo, Istituto Zooprofilattico Sperimentale del Mezzogiorno, Portici, Naples, 80055, Italy; ^2^Istituto Superiore di Sanita, Food Safety, Nutrition, & Veterinary Public Health Department, Rome, 00161, Italy; ^3^Cotugno Hospital, AORN Ospedali dei Colli, Naples, 80131, Italy; ^4^Dipartimento di Medicina Sperimentale, Universita’ degli studi della Campania ‘L. Vanvitelli’, Naples, 80138, Italy; ^5^Department of Medicine, Surgery & Dentistry (Scuola Medica Salernitana), University of Salerno, Baronissi, 84081, Italy

**Keywords:** COVID-19, human-to-animal transmission, interspecies transmission, reverse zoonotic transmission, SARS-CoV-2

## Abstract

SARS-CoV-2, the causative agent of the COVID-19 pandemic, has rarely been associated with transmission from humans to animals (reverse zoonotic transmission). In this retrospective study, the authors reviewed data obtained from 236 animals, including buffaloes, goats/sheep, horses, carrier pigeons, rabbits, hens, snakes, pigs and cows that were screened for SARS-CoV-2 infection because they had been in contact with their SARS-CoV-2-positive breeder for at least 2 weeks. None of the tested animals were found to be positive. The authors' findings suggest that the risk of reverse zoonotic transmission among bred animals and SARS-CoV-2-positive breeders is very low or nonexistent. Additional studies are warranted.

## Lay abstract

SARS-CoV-2, the causative agent of the COVID-19 pandemic, has rarely been associated with transmission from humans to animals. Here the authors reviewed data regarding molecular test results in a total of 236 animals, which were screened for SARS-CoV-2 infection because they had been in contact with their SARS-CoV-2-positive breeder for at least 2 weeks. None of the tested animals were found to be positive. The authors' findings suggest that the risk that bred animals may be infected by SARS-CoV-2-positive breeders is nonexistent, although additional studies are required to confirm these findings.

Coronaviruses represent a group of single-stranded, positive-sense RNA viruses belonging to the subfamily *Coronavirinae* within the family *Coronaviridae* and the order *Nidovirales* [1]. Coronaviruses are capable of infecting a variety of hosts, including; cattle, dogs, pigs, cats, horses, rats, turkeys and humans [2]. In humans, coronaviruses are frequently responsible for the common cold [3], although over the past two decades they have caused three major outbreaks of respiratory syndromes associated with considerable morbidity and mortality rates. Although outbreaks caused by SARS coronavirus 1 in 2004 and MERS coronavirus in 2012 remained confined to the local level, the third outbreak, caused by SARS-CoV-2, quickly turned into a pandemic after the first COVID-19 cases were officially reported in December 2019 in Wuhan (Hubei, China) [4]. As of 24 January 2021, SARS-CoV-2 has infected over 97 million people, with over 2 million deaths worldwide [5]. COVID-19 is considered a zoonotic disease, as SARS-CoV-2 and bat coronavirus RaTG13 share 96.2% of their genetic sequences [6], and the first human cases were epidemiologically linked to wet markets, where different species of live animals, including bats, are kept together in cages in suboptimal hygienic conditions [7]. Although human-to-human transmission is by far the major route responsible for COVID-19 spreading worldwide, several cases of probable reverse zoonotic transmission involving pets, such as cats and dogs, as well as captive animals, such as great apes, lions, tigers and snow leopards, have been reported [8]. Among farmed animals, mink are highly susceptible to SARS-CoV-2 infection [9], whereas the risk of human-to-animal transmission is low in rabbits and nonexistent in other farmed livestock animals according to the World Organisation for Animal Health, which recommends that SARS-CoV-2-positive workers keep away from the animals [10].

The Zooprophylactic Institute of Southern Italy is a public health institution that represents the regional veterinary health authority of Campania and Calabria, with headquarters in Portici (Naples, Italy). The Zooprophylactic Institute of Southern Italy has tested tens of thousands of citizens of the Campania Region and dozens of animals during the pandemic within a mass COVID-19 surveillance program [11]. In this retrospective study, the authors focused attention on animal keepers who noted they had continued to take care of animals after testing positive for SARS-CoV-2. Available test results in animals known to have been in contact with their SARS-CoV-2-positive breeder for at least 2 weeks are reported in this study.

## Methods

In this retrospective study, the authors first identified breeders/keepers who had been in contact with farmed animals for at least 2 weeks after testing positive for SARS-CoV-2 on reverse transcription PCR (RT-PCR). Farmed animals possibly exposed to SARS-CoV-2 via their breeder/keeper could be included in this retrospective study on the condition that the following criteria were simultaneously met:

- Animals had to be bred for commercial purposes;
- Animals had to be in contact with their SARS-CoV-2-positive breeder on a daily basis for an ‘exposure period’ lasting at least 2 weeks;
- The breeder had to test positive for SARS-CoV-2 on RT-PCR performed using nasal and/or buccal swabs at the beginning and end of the ‘exposure period’;
- Animals had to be tested for SARS-CoV-2 with RT-PCR using nasal and/or buccal swabs within 1 week after the exposure period.

SARS-CoV-2 testing using RT-PCR was performed according to WHO guidelines [12] adapted according to the sample assessed (pharyngeal or nasal swab, feces, milk).

## Results

Eighteen different breeders/keepers from 18 different breeding farms were first identified, and a total of 236 animals bred for commercial purposes were included in this retrospective study. The study sample included 65 buffaloes, 27 goats/sheep, 34 horses, ten carrier pigeons, seven rabbits, 21 hens, 12 snakes, 44 pigs and 16 cows. All animals had been in contact on a daily basis with their keeper after he or she tested positive for SARS-CoV-2. The exposure period varied between 14 and 21 days. As requested per study design, the breeder was confirmed to be positive at the end of the exposure period. Nasal and pharyngeal swabs were collected from the animals assessed for SARS-CoV-2 by a veterinary doctor. Feces and, in some cases, milk were also assessed for SARS-CoV-2. Details of the study sample are reported in Table 1.

**Table 1.** Characteristics of the animals tested at 18 different farms.

Animals		Province	n	Housing	Nasal swab (yes/no)	Nasopharyngeal swab	Milk	Feces
Snakes	*Lampropeltis spp., Elaphe spp., Eryx spp., *	Salerno	12	Tank	No	Yes	No	Yes
Horses	Italian Trotter	Salerno	20	Stable/paddock	Yes	Yes	No	Yes
Horses	Italian Trotter	Salerno	14	Stable/paddock	Yes	Yes	No	Yes
Buffaloes	Italian Mediterranean	Caserta	20	Paddock	Yes	Yes	Yes	Yes
Buffaloes	Italian Mediterranean	Caserta	23	Paddock	Yes	Yes	Yes	Yes
Buffaloes	Italian Mediterranean	Caserta	22	Paddock	Yes	Yes	Yes	Yes
Hens	ISA brown	Salerno	11	Battery cage	No	Yes	No	Yes
Hens	Leghorn	Salerno	10	Free	No	Yes	No	Yes
Carrier pigeons		Naples	10	Cage	No	Yes	No	Yes
Rabbits	Half-breed	Salerno	7	Cage	No	Yes	No	Yes
Cows	Italian Friesian	Salerno	10	Stable	Yes	Yes	Yes	Yes
Cows	Italian Friesian	Salerno	6	Stable	Yes	Yes	No	Yes
Goats	Half-breed	Salerno	6	Stable/semi-free	Yes	Yes	No	Yes
Goats and sheep	Half-breed	Salerno	11	Stable/semi-free	Yes	Yes	Yes	Yes
Sheep	Half-breed	Salerno	10	Stable/semi-free	Yes	Yes	No	Yes
Pigs	Duroc, large white	Salerno	15	Pigsty	Yes	Yes	No	Yes
Pigs	Duroc, large white	Salerno	9	Pigsty	Yes	Yes	No	Yes
Pigs	Duroc, large white	Avellino	20	Pigsty	Yes	Yes	No	Yes

Each row represents a single different farm. In each farm, a single keeper/breeder was known to be positive for SARS-CoV-2 and to be in contact with the bred animals.

A total of 168 nasal swab, 236 pharyngeal swab, five milk and 18 feces samples were collected. None of the samples assessed using RT-PCR tested positive for SARS-CoV-2.

## Discussion

Despite advances in the diagnosis, prevention and treatment of COVID-19, the high mutation rate of SARS-CoV-2 and the pandemic proportion of the infection make it difficult to foresee the eradication of such a deadly disease [13]. Although the zoonotic nature of COVID-19 is supported by several findings, the true risk associated with reverse zoonotic transmission remains unknown [14]. Among farmed animals, mink are among the few known to be highly susceptible to SARS-CoV-2 infection. In Denmark, from 15 June 2020 to 4 November 2020, a total of 215 farms were reported to have infected mink, and in two-thirds of the farms assessed, the virus was detected in the totality of the mink [15]. Although some positive dogs and cats were found in this study, none of the rabbits, chickens or horses from the investigated farms tested positive for SARS-CoV-2. These findings support the conclusions of the World Organisation for Animal Health, which considers the risk of reverse zoonotic transmission of SARS-CoV-2 to be high in mustelids, including mink and ferrets, and racoon dogs, low in rabbits and nonexistent in other farmed animals [10]. Nevertheless, the risk of reverse zoonotic transmission may increase with novel SARS-CoV-2 variants. A comprehensive phylogenetic analysis coupled with critical site comparison of the ACE2 receptor showed that several species, including civet, swine, pangolin, cat, cow, buffalo, goat, sheep and pigeon, may be susceptible to SARS-CoV-2 and serve as intermediate hosts [16]. Similar findings have been reported by other authors who have analyzed the amino acid sequences of the ACE2 receptor of a number of wild and domestic animals and concluded that horses, cats, cattle, sheep, European rabbits and grizzly bears may serve as potential SARS-CoV-2 secondary reservoirs [17]. Experimental studies have confirmed that inoculation of SARS-CoV-2 can cause infection of rabbits [18]. Finally, in another *in vitro* study that used respiratory *ex vivo* organ cultures, SARS-CoV-2 was proven capable of infecting the respiratory tissues of sheep and cattle but not of pigs [19]. Furthermore, a SARS-CoV-2 variant capable of replicating more efficiently was also identified, which implies that mutated variants may be more frequently associated with reverse zoonotic transmission.

The authors acknowledge that this retrospective study presents multiple limitations. First, the bred animals tested for SARS-CoV-2 were tested on a single occasion and not retested. Second, animals were tested using only RT-PCR, without performing any serological tests. Third, data regarding duration of contact of breeders/keepers with bred animals after testing positive for SARS-CoV-2 were not retrieved using validated source documents (e.g., registries) but were assessed by interview. Fourth, the sample size was relatively small. Nevertheless, in spite of its multiple caveats, the authors believe this work adds valuable evidence to the existing literature, as it not only confirmed that a large sample of farmed animals were not infected by their positive keeper/breeder but also documented how keepers/breeders did not self-isolate at home and continued to take care of the animals. Positive breeders/keepers should self-isolate at home until they test negative for SARS-CoV-2 as required by local laws and as recommended by the World Organisation for Animal Health. The possibility that novel variants may be associated with more frequent reverse zoonotic transmission cannot be excluded at the present time.

## Conclusion

The findings presented here support the notion that the risk of reverse zoonotic transmission among bred animals and SARS-CoV-2-positive breeders is very low or nonexistent. Additional studies in larger samples both in an experimental and non-experimental setting are warranted.

## Future perspective

Further studies are required to assess the risk of reverse zoonotic transmission among animals and breeders. In particular, data regarding serological responses are needed to explore the risk of reverse zoonotic transmission across different species. Experimental infection of animals would also clearly provide valuable information regarding species susceptibility to SARS-CoV-2, but it would not necessarily reflect the risk of reverse zoonotic transmission in a real-world scenario.

**Summary points**

- Although COVID-19 can be considered a zoonosis, reverse zoonotic transmission is possible, with a limited number of species, such as mink, showing high susceptibility to SARS-CoV-2.
- Eighteen different breeders/keepers from 18 different breeding farms were first identified, and a total of 236 animals bred for commercial purposes, including 65 buffaloes, 27 goats/sheep, 34 horses, ten carrier pigeons, seven rabbits, 21 hens, 12 snakes, 44 pigs and 16 cows, were tested for SARS-CoV-2. The exposure period varied between 14 and 21 days. As requested per study design, the breeder was confirmed to be positive at the end of the exposure period.
- None of the tested animals were positive for SARS-CoV-2 on reverse transcription PCR.
- Although the risk of reverse zoonotic transmission among breeders/keepers and farmed animals is confirmed to be negligible by this study, the possibility that novel variants may be associated with more frequent reverse zoonotic transmission cannot be excluded at the present time.
